# Draft Genome Sequences of 40 *Salmonella enterica* Serovar Typhimurium Strains Isolated from Humans and Food in Brazil

**DOI:** 10.1128/genomeA.00892-16

**Published:** 2016-09-22

**Authors:** Fernanda Almeida, Marta Inês Cazentini Medeiros, Dália Prazeres Rodrigues, Justin Payne, Ruth E. Timme, Marc W. Allard, Juliana Pfrimer Falcão

**Affiliations:** a Universidade de São Paulo- Faculdade de Ciências Farmacêuticas de Ribeirão Preto, Departamento de Análises Clínicas, Toxicológicas e Bromatológicas, Ribeirão Preto, São Paulo, Brazil; b Centro de Laboratório Regional de Ribeirão Preto, Instituto Adolfo Lutz, Ribeirão Preto, Sao Paulo, Brazil; c Laboratório de Enterobactérias, FIOCRUZ/Fundação Instituto Oswaldo Cruz, Rio de Janeiro, Brazil; d Office of Regulatory Science, Center for Food Safety and Applied Nutrition, Food and Drug Administration, College Park, Maryland, USA

## Abstract

Salmonellosis is an important health problem worldwide and *Salmonella enterica* serovar Typhimurium is one of the most common isolated serovars. Here, we reported the draft genomes of 40 *S*. Typhimurium strains isolated from humans and food in Brazil. These draft genomes will improve phylogenetic analysis and will help enhance our understanding of strains of this serovar isolated in Brazil.

## GENOME ANNOUNCEMENT

*Salmonella enterica* is recognized as one of the most common pathogen causes of foodborne disease worldwide (1). In the United States, *S. enterica* causes approximately 1.0 million cases in humans, resulting in nearly 20,000 hospitalizations and more than 378 deaths each year (2). *Salmonella enterica* subsp. *enterica* serovar Typhimurium is one of the most common serovars isolated worldwide, including in Brazil (3).

In this report, we announce 40 draft genome sequences from a collection of *S*. Typhimurium strains isolated from humans and food between 1983 and 2013 from different geographical locations in Brazil.

DNA from each strain was extracted according to published methods (4). Libraries were prepared using 1 ng of genomic DNA with the Nextera XT kit (Illumina, San Diego, CA), and the genomes were sequenced using NextSeq Illumina for 2 × 151 cycles according to the manufacturer’s instructions. *De novo* assemblies were generated from all raw sequence data. The Illumina reads were assembled with SPAdes (5). The contigs for each isolate (draft genomes) were annotated using NCBI’s Prokaryotic Genomes Automatic Annotation Pipeline (PGAAP) (6).

The average G+C mol% of these strains was 52.05%, similar to the reported G+C content for other *Salmonella* strains (7). The genome length was also within the range described for *Salmonella* (4.6 Mb to almost 5.1 Mb) (8). The number of contigs per assembly for each isolate ranged from 47 to 286.

The data provided will help in understanding the molecular epidemiology of *Salmonella* Typhimurium strains isolated from humans and food in Brazil. It will also provide phylogenetic insights into their evolution. A more detailed report of these genomic features will be addressed in a future publication.

### Accession number(s).

The draft genome sequences for these 40 *Salmonella* Typhimurium isolates are available in GenBank and are listed in Table 1.

**TABLE 1:** Metadata for *Salmonella* Typhimurium strains isolated from humans and food between 1983 and 2013 in Brazil

CFSAN no.^*a*^	Isolate name	WGS accession no.^*b*^	Source	Year of isolation	State
CFSAN033848	STm01	LVHC00000000	Human feces	1983	SP
CFSAN033851	STm04	LVGZ00000000	Human feces	1983	SP
CFSAN033852	STm05	LVGY00000000	Human feces	1983	SP
CFSAN033859	STm12	LUJG00000000	Human feces	1984	SP
CFSAN033865	STm18	LVGN00000000	Human feces	1985	SP
CFSAN033866	STm19	LVGM00000000	Human feces	1986	SP
CFSAN033875	STm28	LUJE00000000	Human feces	1988	SP
CFSAN033876	STm29	LVGE00000000	Human feces	1989	SP
CFSAN033877	STm30	LVGD00000000	Human feces	1990	SP
CFSAN033878	STm31	LUJD00000000	Human feces	1991	SP
CFSAN033881	STm34	LVGA00000000	Human feces	1993	SP
CFSAN033882	STm35	LVFZ00000000	Human feces	1995	SP
CFSAN033883	STm36	LVFY00000000	Cold chicken	1995	SP
CFSAN033884	STm37	LVFX00000000	Raw pork sausage	1996	SP
CFSAN033885	STm38	LUJC00000000	Human feces	1997	SP
CFSAN033886	STm39	LUJB00000000	Human feces	1998	SP
CFSAN033887	STm40	LUJA00000000	Lettuce	1998	SP
CFSAN033889	STm42	LUIZ00000000	Human feces	1999	SP
CFSAN033890	STm43	LVFV00000000	Human feces	2000	SP
CFSAN033891	STm44	LVFU00000000	Blood	2000	SP
CFSAN033892	STm45	LUIY00000000	Raw pork sausage	2000	SP
CFSAN033893	STm46	LVFT00000000	Raw Tuscan sausage	2002	SP
CFSAN033894	STm47	LUIX00000000	Human feces	2003	SP
CFSAN033895	STm48	LUIW00000000	Brain abscess	2005	SP
CFSAN033896	STm49	LVFS00000000	Human feces	2010	SP
CFSAN033898	12288/06	LUIV00000000	Swine	2006	SC
CFSAN033904	5937/06	LUIQ00000000	Cold chicken	2006	SC
CFSAN033913	16240/09	LUIJ00000000	Ready-to-eat dish	2009	MS
CFSAN033914	16202/09	LUII00000000	Industrialized product	2009	RS
CFSAN033918	9461/10	LUIF00000000	In natura meat	2010	SC
CFSAN033921	3057/10	LUID00000000	Frozen chicken carcass	2010	PR
CFSAN033922	6346/10	LUIC00000000	Chicken	2010	SP
CFSAN033923	5635/10	LVFL00000000	Unknown (food)	2010	RS
CFSAN033924	9109/10	LVFK00000000	Swine	2010	PR
CFSAN033925	426/10	LUIB00000000	Chicken	2010	SC
CFSAN033928	6709/11	LVFJ00000000	Cold chicken	2011	RS
CFSAN033929	948/12	LUHY00000000	Raw salad	2012	BA
CFSAN033930	1103/12	LUHX00000000	Swine (homemade salami)	2012	RS
CFSAN033932	3330/12	LUHW00000000	Roast beef	2012	SC
CFSAN033933	994/13	LUHV00000000	Final product sales (animal origin)	2013	SP

a CFSAN, Center for Food Safety and Applied Nutrition.

b WGS, Whole-Genome Shotgun.

## References

[1] Majowicz SE, Musto J, Scallan E, Angulo FJ, Kirk M, O’Brien SJ, Jones TF, Fazil A, Hoekstra RM; International Collaboration on Enteric Disease “Burden of Illness” Studies 2010 The global burden of nontyphoidal Salmonella gastroenteritis. Clin Infect Dis 50:882–889. doi:10.1086/650733. 20158401

[2] Scallan E, Hoekstra RM, Angulo FJ, Tauxe RV, Widdowson MA, Roy SL, Jones JL, Griffin PM 2011 Foodborne illness acquired in the United States—major pathogens. Emerg Infect Dis 17:7–15. doi:10.3201/eid1701.091101p1. 21192848 10.3201/eid1701.P11101 PMC3375761

[3] Hendriksen RS, Vieira AR, Karlsmose S, Lo Fo Wong DM, Jensen AB, Wegener HC, Aarestrup FM 2011 Global monitoring of *Salmonella* serovar distribution from the World Health Organization global Foodborne Infections Network Country Databank: results of quality assured laboratories from 2001 to 2007. Foodborne Pathog Dis 8:887–900. doi:10.1089/fpd.2010.0787. 21492021

[4] Campioni F, Falcão JP 2014 Genotypic diversity and virulence markers of Yersinia enterocolitica biotype 1A strains isolated from clinical and nonclinical origins. APMIS 122:215–222. doi:10.1111/apm.12126. 23763723

[5] Nurk S, Bankevich A, Antipov D, Gurevich AA, Korobeynikov A, Lapidus A, Prjibelski AD, Pyshkin A, Sirotkin A, Sirotkin Y, Stepanauskas R, Clingenpeel SR, Woyke T, McLean JS, Lasken R, Tesler G, Alekseyev MA, Pevzner PA 2013 Assembling single-cell genomes and mini-metagenomes from chimeric MDA products. J Comput Biol 20:714–737. doi:10.1089/cmb.2013.0084. 24093227 PMC3791033

[6] Klimke W, Agarwala R, Badretdin A, Chetvernin S, Ciufo S, Fedorov B, Kiryutin B, O’Neill K, Resch W, Resenchuk S, Schafer S, Tolstoy I, Tatusova T 2009 The National Center for Biotechnology Information’s protein Clusters Database. Nucleic Acids Res 37:D216–D223. doi:10.1093/nar/gkn734. 18940865 PMC2686591

[7] Papanikolaou N, Trachana K, Theodosiou T, Promponas VJ, Iliopoulos I 2009 Gene socialization: gene order, GC content and gene silencing in salmonella. BMC Genomics 10:597. doi:10.1186/1471-2164-10-597. 20003346 PMC2801525

[8] Cao G, Meng J, Strain E, Stones R, Pettengill J, Zhao S, McDermott P, Brown E, Allard M 2013 Phylogenetics and differentiation of salmonella Newport lineages by whole genome sequencing. PLoS One 8:e55687. doi:10.1371/journal.pone.0055687. 23409020 PMC3569456

